# Enhancing clinical service design for multimorbidity management: A comprehensive approach to joined‐up care for diabetes, chronic kidney disease, and heart failure

**DOI:** 10.1111/dme.15403

**Published:** 2024-07-08

**Authors:** Saif Al‐Chalabi, Smeeta Sinha, Philip A. Kalra

**Affiliations:** ^1^ Donal O'Donoghue Renal Research Centre, Salford Royal Hospital Northern Care Alliance NHS Foundation Trust Salford UK; ^2^ Faculty of Biology, Medicine and Health University of Manchester Manchester UK; ^3^ Manchester Academic Health Science Centre University of Manchester Manchester UK

**Keywords:** cardiovascular‐kidney‐metabolic syndrome, chronic kidney disease, diabetes mellitus, heart failure, integrated care, multimorbidity, telehealth

## Abstract

**Background and Aims:**

Multimorbidity is becoming the norm rather than the exception, especially among the ageing population and people with lower socio‐economic status. In addition to the rising healthcare cost, multimorbidity poses considerable difficulty in the delivery of adequate holistic care for affected patients.

**Methods:**

This review presents a discussion of the current barriers to delivering holistic care to people with multimorbidity and proposes a model of clinical care for people living with cardiovascular‐kidney‐metabolic (CKM) syndrome as an exemplar of a multimorbidity cluster.

**Results:**

Single organ/disease services may not be able to provide optimum care to people with multimorbidity due to the potential complex interactions between multiple disease symptoms and management. In addition, people with multimorbidity may be required to attend multiple appointments in different healthcare centres. This may negatively impact access to services due to time and financial burden. Other barriers include co‐ordinating communication between healthcare professionals and reduced continuity of care. Optimising CKM health requires patient‐centred care led by an interdisciplinary care team who ideally should possess CKM competencies utilising a shared care protocol to coordinate evidence‐based care and use of telehealth to empower patients. Stakeholders and policymakers need to adapt new policy models to establish and enhance CKM care models by allocating funds and implementing frameworks for educational reforms.

**Conclusions:**

A CKM service has the potential to increase the uptake of cardiac and renal protective medications as well as optimising metabolic care, increase capacity in both primary and secondary care, improve quality of life and clinical outcomes, reduce patient inconvenience, and importantly allow rapid translation of advances in cardiorenal metabolic diseases into clinical practice.

## Graphical abstract

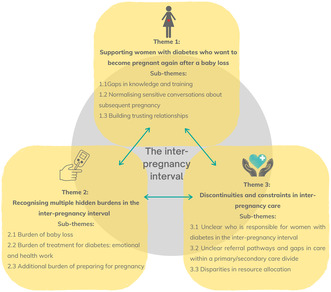

**What's new?**

- People with multiple conditions are more likely to have worse clinical outcomes. Barriers to providing optimum care include interaction between multiple clinical specialties, difficulty in accessing care, reduced continuity of care and ineffective communication and inadequate skill mix between healthcare professionals.
- Optimising care for people with diabetes, chronic kidney disease, and heart failure requires patient‐centred care led by an interdisciplinary team utilising a shared protocol encompassing evidence‐based guidelines and use of telehealth to empower patients and reduce health inequalities.
- This has the potential to improve outcomes, reduce patient inconvenience, and allow rapid translation of advances in research into clinical practice.

## 1 INTRODUCTION

For the last two decades, there has been an increase in the interest in multimorbidity, commonly defined as the presence of two or more long‐term conditions in an individual. This is owing to the mounting evidence, showing people with multimorbidity are more likely to have worse quality of life and clinical outcomes compared to people with a single long‐term condition.^1^ Multimorbidity is becoming the norm rather than the exception, especially among the ageing population, and people with lower socio‐economic status (SES). A study of 1.75 million people living in Scotland found that 23% had multimorbidity.^2^ Those living in the most deprived areas of the UK are most affected with earlier onset of multimorbidity by 10–15 years when compared to people living in the least deprived areas. Similar figures were also observed in a US‐based study where 21% of Americans had multimorbidity. The estimated care cost for these people was more than three‐quarters of the total health expenditure of the United States.^3^ In addition to the rising prevalence of multimorbidity and its associated healthcare costs, there is considerable difficulty in delivering adequate holistic care for affected patients. This is due to the interacting pathologies between multiple conditions, which necessitate careful consideration of treatment strategies to avoid adverse outcomes.

Multimorbidity is a broad term and may not necessarily be helpful in structuring clinical care services because it involves any combination of two or more conditions with a wide spectrum of varying degrees of severity. For example, the combination of mild controlled asthma and osteoporosis may not have the same impact on quality of life and risk of death in a patient when compared to someone with advanced chronic kidney disease (CKD), severe heart failure (HF), and diabetes mellitus (DM). Therefore, identifying common disease clusters could provide a more meaningful way to design and deliver a bespoke care plan. Growing evidence of the interrelated pathophysiological pathways between CKD, cardiovascular diseases (CVD), and DM has led to the conceptualisation of the cardiovascular‐kidney‐metabolic (CKM) syndrome.^4^ There are multidirectional relationships among these conditions, which often coexist in certain populations; for example, people with central obesity will also have common risk factors for CVD such as dyslipidaemia and hypertension. This places an increased burden on patients, as well as health services globally.^5^

The current traditional healthcare model was designed around the management of a single disease/organ rather than managing the individual with their personalised medical problems.

In addition, the increasing proportion of patients with multimorbidity and the significant UK National Health Service (NHS) treatment backlog have encouraged clinical services to develop new models of care, which aim to deliver benefits at both organisational and patient levels.^6^

This review presents a discussion of the current barriers to delivering holistic care to people with multimorbidity. It is aimed at healthcare professionals and policymakers to propose a clinical service design that coordinates care for people living with CKM syndrome as an exemplar of more optimised management of a multimorbidity cluster.

## 2 BARRIERS TO OPTIMISE CARE FOR PEOPLE WITH MULTIMORBIDITY

### 2.1 Complexity of multimorbidity

Multiple conditions in a single individual may increase the difficulty of providing optimum care by healthcare professionals who are used to providing a single organ/disease service. Important clinical interactions are prone to happen between multiple diseases in terms of symptoms and management.^7^ For example, prescribing mineralocorticoid receptor antagonists (MRA) to a patient with heart failure may lead to worsening kidney function and hyperkalaemia.^8^ As a consequence, patients might be deprived of the benefits of these cardioprotective therapies. Another example includes people with asthma who may experience difficulty in taking regular exercise to reduce weight and improve their cardiovascular health. From patients' perspectives, multimorbidity can also affect their understanding of medical conditions and their management due to extended, complex, and sometimes counteractive explanations provided for their different conditions.^9^ This may influence access to care and may result in patients missing healthcare appointments due to a lack of understanding of the reason for attendance and misunderstanding of priorities.

### 2.2 Access to services

People with multimorbidity are usually required to attend multiple appointments in different healthcare centres, which are often specialty specific. This is time‐consuming for patients and their carers. Furthermore, attendance may be negatively impacted due to financial constraints, for example the cost of transport to attend multiple clinics.^7^ This could be a factor as to why people with multimorbidity and lower SES have poor access to healthcare services, which in turn leads to poorer health.

### 2.3 Communication between healthcare professionals

Interprofessional communication is central to delivering care for people with multimorbidity. Evidence shows that good interprofessional communication improves the quality of life and reduces the risk of death in these complex patients.^10^, ^11^ Poor communication between healthcare professionals has a higher impact on people with multimorbidity. Conflicting advice might arise from different clinical visits to different organ‐specific providers. For example, a patient with HF and CKD might have different advice from the cardiologist and the nephrologist about the dose of loop diuretics to manage the patient's symptoms of breathlessness due to fluid overload, or use of renin–angiotensin blockade or mineralocorticoid inhibitors when hyperkalaemia supervenes. Another factor may include delays in correspondence between specialists, patients, and the primary care physician (PCP). The current standard method of communication in the NHS is printed letters.^12^ This may take days to weeks to be dictated, typed, corrected, and then sent to the patient's PCP and uploaded to the electronic health record system. Communication delays and conflicting advice have the potential to impact patients' understanding of their complex health needs. This will result in a lack of patient self‐motivation to manage their multiple conditions.

### 2.4 Continuity of care

Continuity of care has been declining in the last 3 decades due to several reasons including reduced numbers of PCPs and the growth of specialisation in medical practice.^13^ People with multimorbidity are increasingly seen by multiple different healthcare professionals, which increase the likelihood of fragmented care.^14^ Policies urge the enhancement of continuity of care to foster patients' empowerment and improve communication and trust between patients and their healthcare professionals.^15^, ^16^, ^17^ Continuity of care builds a strong patient–doctor relationship that allows the healthcare professional to suggest more suitable management plans to suit the patient's lifestyle, physical and mental health, and these suggestions are more likely to be accepted by the patient. This increases both patient and healthcare professional satisfaction due to more knowledge of the patient's personal and clinical history.^18^ Evidence shows that providing a high continuity of care reduces the risk of adverse outcomes such as longer hospital stays and frequent emergency admissions.^19^, ^20^, ^21^ Current models of healthcare focus on continuity of care mainly in primary care where patients may have a named doctor who is responsible for their general healthcare. However, when a patient with multimorbidity is referred to multiple specialists in secondary and tertiary centres, care is diverted to different departments with the PCP being the hub correspondent. This has the potential to reduce the continuity of care due to potentially conflicting advice and delayed communications between different specialty teams, which may result in poorer outcomes.

## 3 PROPOSED ENHANCEMENTS FOR CARDIOVASCULAR‐KIDNEY METABOLIC CARE

### 3.1 Integrated care models

Providing integrated care models in a CKM service is an integral concept to deliver coordinated and comprehensive care. These models have the benefit of simultaneously addressing the complex interaction between elements of the CKM syndrome.

#### 3.1.1 Multiple‐disciplinary teams

Multidisciplinary and interdisciplinary teamwork are increasingly utilised frameworks in modern medicine. Multidisciplinary teams draw on knowledge from different specialities but stay within the boundaries of each specialty, while interdisciplinary teams develop a joint service plan for the patient after discussing each speciality's individual assessment.^22^

Multiple‐disciplinary teamwork is a well‐known and widely applied method to coordinate patients' care and improve their outcomes.^23^, ^24^ An ideal CKM service would comprise a wide array of healthcare professionals including PCPs, specialists in endocrinology, nephrology and cardiology, nurses with special interest in CKM, dieticians, and pharmacists. All members would collaborate as one interdisciplinary team to provide a personalised care plan that addresses all aspects of the patient's CKM health. For example, hospital pharmacists may have a key role in restarting cardiorenal metabolic therapies after their cessation during acute illness. Failure to reintroduce cardio‐and renoprotective agents has been associated with worse outcomes. Recommencement of therapy often gets missed, which may result in adverse outcomes.^25^ Care plans can be devised during combined clinics or via regular face‐to‐face or online meetings. Other aspects of patients' health, such as frailty and mental health, can also be addressed via specialist nurses or other healthcare professionals in frailty and mental health and aligning with local care service design.

#### 3.1.2 Shared care protocols

There are limited shared protocols and guidelines for patients with multiple conditions. A recent Cochrane review concluded that there were limited benefits in clinical outcomes to support shared policies in the management of people with multimorbidity.^26^ This was largely due to the relatively small number of randomised controlled trials conducted to address this research question. However, the management of CKD and DM has gained significant interest in recent years due to the close relationship between the two conditions. Kidney Disease: Improving Global Outcomes (KIDIGO) produced a guideline titled “*Clinical Practice Guideline for Diabetes Management in Chronic Kidney Disease”* in 2020, and it was updated in 2022.^27^ The guideline encouraged healthcare services to adopt team‐based integrated care to manage patients with DM and CKD. It suggested delivering care by an interdisciplinary team of physicians and other supporting healthcare professionals in defined steps: establishing a register with risk assessment, assessing cardiometabolic risk factors and kidney function, reviewing treatment targets, reinforcing self‐management, and providing counselling on diet and exercise.

**FIGURE 1:**
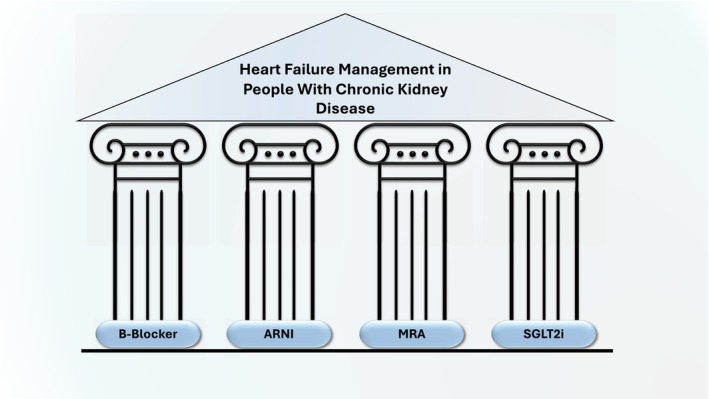
The four pillars of care to manage heart failure in patients with chronic kidney disease. ARNI, angiotensin receptor‐neprilysin inhibitor; MRA, mineralocorticoid receptor antagonists; SGLT2i, sodium‐glucose cotransporter‐2 inhibitors.

In addition, the 2021 European Society of Cardiology (ESC) Guidelines for the diagnosis and treatment of acute and chronic HF referenced the management of concomitant HF and CKD.^28^ It highlighted the need to reduce cardiovascular events in patients with CKD by using the four pharmacological pillars of heart failure treatment: an angiotensin receptor‐neprilysin inhibitor (ARNI), a beta‐blocker, an MRA, and a sodium‐glucose cotransporter‐2 (SGLT2) inhibitor (Figure 1). However, it did not provide specific guidance on introduction, monitoring, and dose adjustments. Recent RCTs have also identified that non‐steroidal MRA and the glucose‐lowering agent glucagon‐like peptide‐1 agonists (GLP1 RAs) can also improve cardiovascular and renal outcomes.^29^, ^30^ Therefore, there is a pressing need to adapt to the rapidly evolving evidence base within CKM care and ensure guidelines and protocols are updated promptly to support front‐line clinicians across multiple specialities. This coupled with local improvement programmes may improve the low uptake of renal and cardiac protective medicines and reduce the burden of morbidity and mortality.^4^, ^31^

Two models of CKM care were suggested by the American Heart Association presidential advisory to reduce fragmented care and enhance a comprehensive approach to patients with CKM syndrome: value‐based and volume‐based.^4^ The former involves the development of protocolised guidance for people with CKM syndrome among PCPs and specialists. This team would be supported by a CKM coordinator to ensure continuity of care between PCPs, subspecialists in CKM and the wider allied healthcare professionals such as pharmacists. Volume‐based care involves targeted referrals of high‐risk patients to subspecialists and as follows: nephrology for advanced CKD, cardiology for patients with prevalent CVD and echocardiography abnormalities, and endocrinology for patients with poorly controlled DM. In both models, potential conflicting advice from different healthcare professionals can be addressed by using case‐based discussions during MDTs.

When designing a new CKM service, it is important to consider the wide spectrum of disease severity in patients with CKM. A service for early diabetic kidney disease in a deprived community may focus on early detection and engagement, while one for advanced kidney disease with severe HF may focus on strategies to improve quality of life and advanced care planning. Additionally, an expanded CKM service may manage related conditions like metabolic dysfunction‐associated steatohepatitis (MASH) and hyperlipidaemia.

Examples of frameworks that target specific groups of patients with CKM syndrome are the joint Association of British Clinical Diabetologists (ABCD) and UK Kidney Association (UKKA) guidelines “*Management of lipids in adults with diabetic kidney disease*” and “*Standards of Care for Glycaemic Assessment in People with Diabetes on Haemodialysis*.”^32^, ^33^ The latter recommended the use of direct glucose monitoring in people with diabetes who were receiving regular haemodialysis. The guideline also highlighted the importance of ensuring renal diabetes MDT input.

Quality improvement projects are an excellent way to test amendments and reshape the service to fit best with the established clinical services. The aim is to establish an agreed protocol among primary and secondary healthcare providers. This should be based on evidence‐based medicine and also adhere to the international best practices for guideline development.^34^

### 3.2 Patient‐centred care

Patient‐centred care (PCC) is a fundamental concept to deliver holistic care to patients with CKM syndrome due to the complex clinical needs of affected patients. It improves clinical outcomes and quality of life in a variety of service settings.^35^ To apply PCC, clinicians should empower patients to identify their goals, values, and priorities by communicating effectively with patients and their carers and avoiding assumptions about people's decisions.

Educational interventions are key to empowering patients and are an integral part of PCC. It allows patients to take control of their conditions by undertaking behavioural changes to improve their well‐being and clinical outcomes.^36^, ^37^, ^38^, ^39^ An important aspect of educating people with CKM syndrome is to align education programmes to prevent the potential confusion that can arise from different speciality‐specific education programmes.

PCC can improve patients' understanding of their complex conditions, which may result in better medication adherence.^40^ Medication non‐adherence is a common issue in healthcare and affects more than 50% of patients.^41^ Patients with CKM may have higher rates of non‐adherence due to the burden of multiple conditions and polypharmacy. To identify patients' focused goals and priorities, standardised questionnaires that focus on patients' experiences and reported outcomes should be undertaken regularly. In addition, less engaged patients can be identified by auditing missed appointments and can be helped by offering mental health support if required and by applying individualised and ethnically appropriate care plans.

### 3.3 Utilising telehealth

Telehealth is the delivery of healthcare services over distance. It has the potential to reduce health inequalities by improving access to services in remote and deprived areas. Virtual surveillance and MDT case reviews are good examples of utilising telehealth, which were supported by a recent statement from the Royal College of Physicians (RCP).^42^ However, services should be designed to ensure face‐to‐face access for vulnerable people with limited digital literacy or access to reduce variation in access and to ultimately provide equity of care.^43^

Effective information technology (IT) systems allow rapid communication between different teams to support timely decisions and ensure PCC. Timely identification of significant changes in metrics such as HbA1c and estimated glomerular filtration rate (eGFR) will allow accurate coding of chronic conditions such as DM and CKD. In addition, it will trigger care pathways that ensure regular monitoring, early treatment and risk identification by using tools such as the kidney failure risk equation (KFRE).^44^ There is a significant variation of coding of conditions among primary care practices, which poses a major issue that halts effective management of chronic conditions.^45^

Telehealth has been shown to be effective across a wide range of medical disciplines.^46^ It may enhance the management of patients with CKM syndrome by improving access to care, facilitating communication among healthcare providers, and enabling remote monitoring of important parameters that can be shared between healthcare professionals. This can reduce unnecessary clinical visits and provide more timely care for patients. Conducting telephone or video consultations might be more convenient for some patients. It also enables more efficient allocation of the limited face‐to‐face consultations to be used for patients with more complex clinical needs. Telemedicine interventions have been shown to improve important CKM clinical parameters such as blood sugar and blood pressure.^47^, ^48^ It has also been shown to improve clinical outcomes such as with reduced major thromboembolic events in patients with CVD and reduced HF‐related hospital admissions and mortality.^49^, ^50^

The WHO suggested an 11‐step plan to implement telehealth intervention.^51^ These steps are categorised into three phases, situational assessment, implementation planning, and monitoring, evaluation, and continuous improvement.

### 3.4 Policy models

**FIGURE 2:**
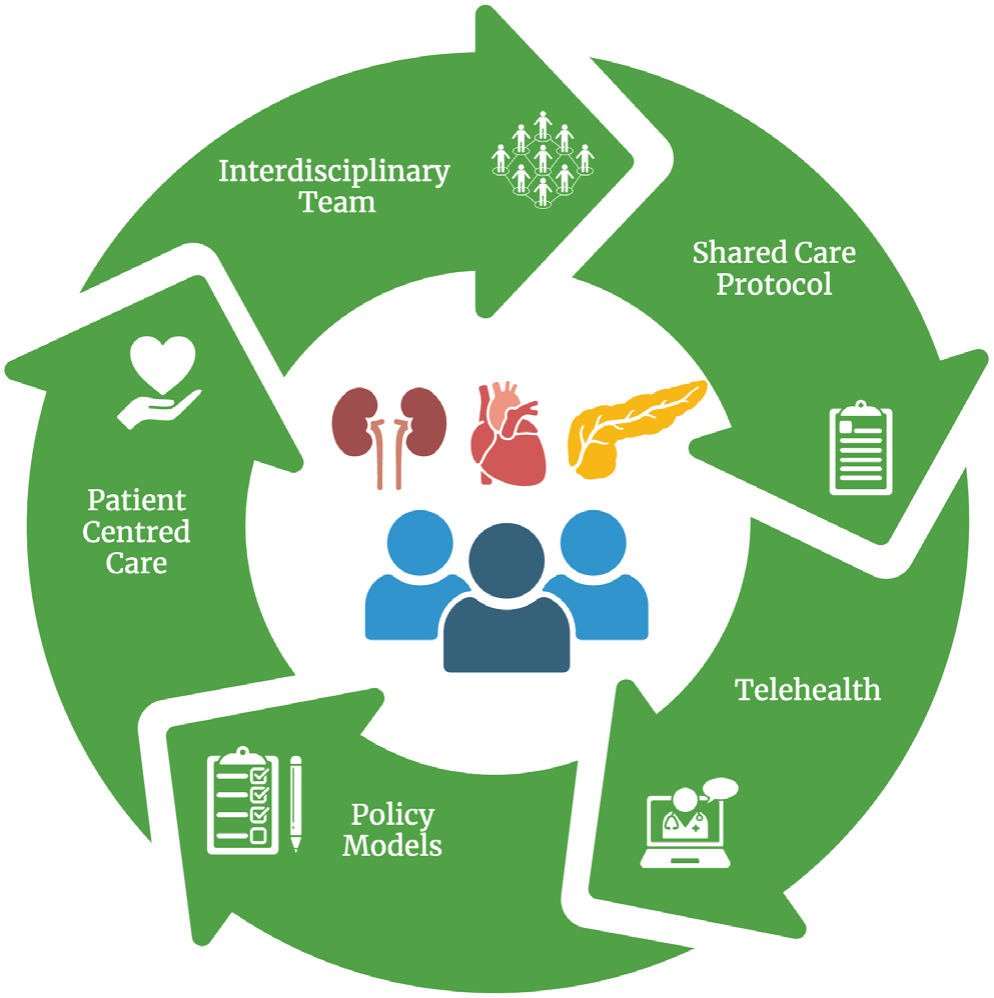
Proposed enhancements for cardiovascular‐kidney metabolic care.

The proposed enhancements for joined‐up care will not thrive without adapting new policy models by the health policymakers (Figure 2). In recent years, there has been a general move towards integrated care. The NHS long‐term plan published in 2019 supported the application of integrated care models.^52^ Since July 2022, England has formally divided into 42 area‐based integrated care systems (ICSs). The boards of these integrated care systems are responsible for the delivery of equitable care across their populations with a view to improving health outcomes. Project INTEGRATE is underway to examine and develop integrated care models to support European care systems to respond to the challenges of the ageing population and the epidemic of long‐term conditions.^53^ The aim is to provide managerial and policy recommendations to ultimately develop a European policy model for integrated care.

Addressing the transformation to integrated care models requires health managers to advocate policies that support its development and funding. This requires movement away from traditional frameworks for quality improvement to adopt a strategic organisational approach that focuses on patients rather than a single disease/organ and dissolves the classic current divide between focused services. This will allow funding to flow from individual services and institutions towards services with shared goals. Funding can be used to develop interdisciplinary teams, telehealth services, and patient self‐management programmes. One of the ways to divert more funding to these care models is to fundamentally change the predetermined set of outcomes from disease‐specific to patient‐centred outcomes. This can be done by creating incentive structures for healthcare providers that reward the delivery of high‐quality, coordinated care. This could include performance‐based reimbursements that consider patient outcomes, satisfaction, and reductions in hospital readmissions.

To deliver high‐quality CKM services, new policies are needed to implement new regulatory frameworks for educational and professional reforms. This will ensure adequate CKM competencies among healthcare professionals providing CKM services. One suggestion to provide these competencies is to develop a new CKM subspecialty for those doctors who have completed their training in cardiology, nephrology, or endocrinology. Alternatively, CKM competencies might be incorporated within the training programmes of these specialties. Other healthcare professionals, including established consultants, may develop CKM competencies via a similar training programme with modular courses.

## 4 POTENTIAL BENEFITS OF A CKM SERVICE

1. Increase the uptake of cardiorenal protective medications such as SGLT2 inhibitors and GLP1 RAs.
2. Facilitate renin–angiotensin–aldosterone system inhibitor (RAASi) optimisation by controlling RAASi‐related hyperkalaemia using potassium binders and better understanding of eGFR changes.
3. Increase capacity in both primary and secondary care by reducing the total number of clinic appointments needed for each patient or by offering alternative models of care such as virtual surveillance and MDT case reviews.
4. Patient empowerment by enhancing patient‐centred care and provision of continuity of care.
5. Improve patients' quality of life by reducing variance of care and frequent and multiple clinic visits.
6. Rapid translation of advances in cardiorenal metabolic diseases into clinical practice by multi‐specialty experts.
7. Development of a cadre of clinicians with multi‐specialty skills.

## 5 CONCLUSION

Cardiovascular, metabolic, and kidney diseases often coexist and share common pathophysiological pathways. This overlap is now described as CKM syndrome. Optimising CKM health requires patient‐centred care led by an interdisciplinary care team with CKM competencies utilising a shared care protocol to coordinate care and use of telehealth to empower patients. Stakeholders and policymakers need to adapt new policy models to establish and enhance CKM care models by allocating funds and implementing frameworks for educational reforms. The transformation to this model of care from the current siloed practice could be challenging. However, healthcare professionals—both in primary and secondary care—are encouraged to focus their efforts and collaborate to overcome the obstacles in adopting this new model of CKM care. A CKM service has the potential to increase the uptake of cardio and renal protective medications, increase capacity for patient consultations in both primary and secondary care, improve quality of life and clinical outcomes, reduce patient inconvenience, and importantly allow rapid translation of advances in cardiorenal metabolic diseases into clinical practice.

## FUNDING INFORMATION

The authors have no funding sources to declare.

## CONFLICT OF INTEREST STATEMENT

Professor Sinha reports grants from AstraZeneca, Johnson & Johnson, and Amgen; consulting fees from CSL Vifor and Inozyme Pharma; honoraria from Bayer, Menarini, AstraZeneca, GSK, Novartis, Sanofi‐Genzyme, Boehringer Ingelheim, CSL Vifor, and Medscape; and support for attending meetings from AstraZeneca, Novartis, and CSL Vifor. Professor Sinha is the NHS England National Clinical Director for Renal Medicine. Professor Kalra reports research grant support from CSL Vifor and Astellas; consulting fees from AstraZeneca, CSL Vifor, Unicyte, and UCB; honoraria from CSL Vifor, AstraZeneca, Bayer, Pharmacosmos, GSK and Pfizer; and support to attend meetings from Pharmacosmos and CSL Vifor. Dr Al‐Chalabi has no conflict of interest to declare.

## Data Availability

Data sharing is not applicable to this article as no new data were created or analysed in this study.
